# Association and impact of inflammatory markers and cardiac structure on atrial fibrillation risk: a study integrating NHANES with real-world data

**DOI:** 10.3389/fcvm.2025.1724217

**Published:** 2026-01-13

**Authors:** Dingbang Wang, Guofeng Zhou, Jiahui Liu, Yu Xiu, Haiying Li, Bo Li

**Affiliations:** 1 School of Clinical Medicine, Shandong Second Medical University, Weifang, China; 2 Department of Cardiology, Zibo Central Hospital, Zibo, China; 3 Medical Integration and Practice Center, Cheeloo College of Medicine, Shandong University, Jinan, China; 4 Department of Ultrasound, Zibo Central Hospital, Zibo, China; 5 Human Resources Department, Zibo Central Hospital, Zibo, China

**Keywords:** atrial fibrillation, left atrial diameter, NHANES, system inflammation response index, systemic inflammation markers

## Abstract

**Background:**

Inflammation plays a central role in the pathogenesis of atrial fibrillation (AF), a common cardiac arrhythmia. Complete blood count (CBC)-derived markers of inflammation, including the systemic inflammatory index (SII) and systemic inflammatory response index (SIRI), have emerged as novel biomarkers of systemic inflammation. Although small prior studies have reported associations between certain inflammatory markers and AF, their limited sample sizes and potential baseline imbalances prevent definitive conclusions. Using a large population-based cohort, this study examines the association between CBC-derived inflammatory markers—SIRI, SII, monocyte-to-lymphocyte ratio (MLR), aggregate index of systemic inflammation (AISI), neutrophil-monocyte-to-lymphocyte ratio (NMLR), neutrophil-to-lymphocyte ratio (NLR), and platelet-to-lymphocyte ratio (PLR)—and the risk of AF, and investigates potential mediating mechanisms by integrating clinical data.

**Methods:**

This cross-sectional analysis included 10,474 adults aged ≥20 years from the National Health and Nutrition Examination Survey (NHANES) 2013–2020 and was validated in 13,707 adults aged ≥20 years from Zibo Central Hospital 2015–2024. Seven inflammatory markers were derived from CBC data and categorized into low, medium, and high quartiles according to their distributions. A weighted multivariable logistic regression adjusted for confounders, including age, sex, hypertension, and diabetes. Associations between inflammatory markers and atrial fibrillation were expressed as odds ratios (OR) with 95% confidence intervals (CI). Restricted cubic spline (RCS) regression assessed nonlinear relationships. Subgroup and interaction analyses evaluated the influence of demographic and clinical factors. Finally, a mediation model examined the mediating role of left atrial diameter.

**Results:**

Of the 10,474 participants in the NHANES database, 136 (1.3%) were diagnosed with AF. After full adjustment, the highest SIRI tertile showed a significantly increased risk of AF compared with the lowest tertile (OR = 2.182; 95% CI: 1.094–4.354; *P* = 0.027). RCS analysis revealed a linear positive association between SIRI and AF risk (overall P > 0.05). Subgroup analyses and interaction tests indicated that the positive association between SIRI and AF persisted across different conditions (all *p*-value for interaction > 0.05). Results were then validated using the case management system of Zibo Central Hospital. In that cohort, after full adjustment, the highest SIRI tertile again had a significantly increased risk of AF vs. the lowest tertile (OR = 1.436; 95% CI: 1.248–1.652; *P* < 0.001). Mediation analysis indicated that LA diameter mediated 13.54% of the association between SIRI and AF.

**Conclusion:**

This study suggests that elevated SIRI may represent a potential biomarker and is associated with an increased risk of AF, and found that left atrial diameter may partially mediate this relationship, suggesting SIRI as a potential inflammatory biomarker for AF prediction. Although other CBC-derived markers did not show significant associations, the results underscore inflammation's role in AF pathogenesis. Further longitudinal studies are needed to validate these findings and clarify the underlying mechanisms.

## Graphical Abstract

**Figure:**
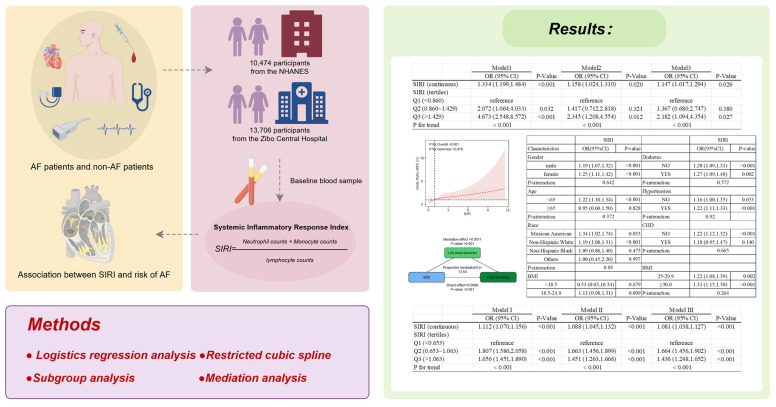
Created using Figdraw.

## Introduction

Atrial fibrillation (AF) is the most common sustained arrhythmia and is linked to higher risks of stroke, heart failure, and death, contributing to rising cardiovascular incidence and mortality (1, 2). The estimated prevalence of AF in adults is 2% to 4%, and its incidence is climbing rapidly, with 12.1 million people projected to be affected by 2030. Prevalence increases with age: it is under 0.5% in the 40–50 age group and reaches 5% to 15% by age 80, and it occurs more often in men than in women. In high-risk patients, the annual risk of thromboembolic stroke can reach 9%, and mortality is doubled (3).

Peripheral blood cells and their inflammatory factors play a crucial role in the occurrence and maintenance of AF and are important therapeutic targets for intervention. Previous studies have shown that macrophages, monocytes, lymphocytes and their products (such as IL-1β, IL-6) IL-8 and TNF-α are involved in the pathogenesis of AF (4–10). Macrophages, monocytes lymphocytes and other immune cells are involved in regulating the differentiation of CD4+ T cells and B cells, promoting the occurrence of local inflammatory response, and inducing the occurrence of AF. In addition, monocytes and macrophages can secrete IL-1 *β* which activates downstream proteins to increase susceptibility to AF after binding to the receptor. In addition, Platelets can also promote the occurrence and maintenance of AF through TGF-*β*-dependent mechanisms (11). Left atrial diameter is an indispensable index to evaluate left atrial function. During atrial fibrillation, abnormal electrical signals in the atrium lead to atrial systolic and diastolic dysfunction, which can cause atrial enlargement in the long term. In addition, left atrial enlargement is also an important risk factor for AF. Long-term hypertension, valvular heart disease and other diseases can lead to increased left atrial pressure or volume load, causing structural and functional changes of atrial myocytes, thereby increasing the risk of atrial fibrillation, forming a vicious circle. Plasma levels of inflammatory markers, such as IL-6, are associated with left atrial diameter and may promote atrial structural remodeling in patients with AF, leading to the occurrence of AF (12, 13). AF is an inflammatory disease and peripheral blood cells and their inflammatory factors play a crucial role in its pathogenesis and progression. However, there is limited research available on the relationship between AF and CBC-derived inflammatory markers.

CBC-derived inflammatory markers include neutrophils, lymphocytes, platelets, and monocytes. These markers are employed to predict outcomes in various inflammation-related diseases. The NLR has shown improved prognostic value for cardiovascular disease (CVD) mortality and related outcomes (14–16). The SII and SIRI are recently proposed composite biomarkers that combine immune cell subsets with platelet counts (17, 18). Researchers have widely applied these indices to examine links between chronic inflammation and diverse conditions such as cancer, metabolic disorders, and other inflammatory diseases (19, 20). Although some studies report associations between SII or SIRI and atrial fibrillation (AF) in stroke patients and in paroxysmal AF (21, 22), the evidence derives mainly from small clinical cohorts, and no definitive conclusion has been reached, underscoring the need for population-level investigation.

The relationships between other CBC-derived inflammatory markers and AF have not been fully evaluated. To our knowledge, the association between CBC-derived inflammatory markers and left atrial diameter has not been clearly established. Therefore, we conducted a cross-sectional study using the National Health and Nutrition Examination Survey (NHANES) database and the inpatient case system of Zibo Central Hospital to further examine these associations. We assessed links between inflammatory markers and AF, investigated associations between additional inflammatory markers and AF, and identified potential mediators of the inflammatory marker–AF relationship using mediation analysis. The primary objective of this study was to assemble evidence on biomarkers that might enable earlier detection of AF.

## Methods

### Data selection and study design

**Figure 1:**
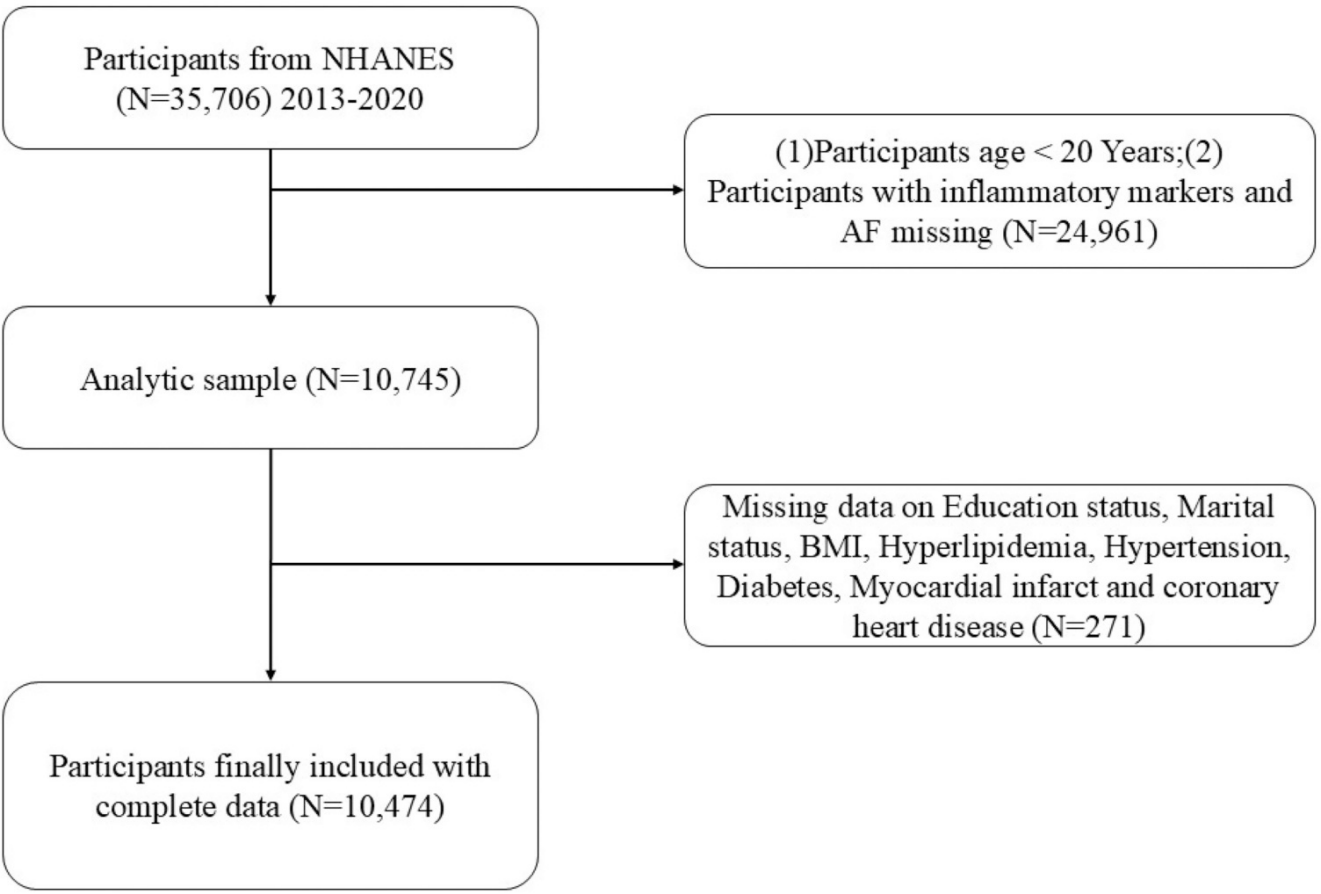
Flowchart of the participant selection from NHANES 2013–2020.

The NHANES employs a complex sampling design to produce a representative sample of the US population every two years. Its primary purpose is to assess the health and nutritional status of Americans. The NHANES program was approved by the Institutional Review Board of the National Center for Health Statistics, and each participant provided written informed consent. The survey includes demographic, dietary, physical examination, laboratory, and questionnaire data. A total of 10,474 participants aged 20 years and older were included across four survey cycles from 2013 to 2020 (Figure 1).

In addition, through the inpatient case management system of Zibo Central Hospital, from 2015 to 2024. Information on eligible participants diagnosed with atrial fibrillation met inclusion criteria and after exclusion criteria, a total of 13,707 patients aged 20 years or older were included. Exclusion criteria included a history of congenital heart disease, valvular heart disease, left ventricular systolic dysfunction, cardiomyopathy, previous cardiac surgery, thyroid disease, recent infection, autoimmune or inflammatory diseases, and malignant tumors with a life expectancy shorter than 1 year or those suffering from other end-stage diseases.

### The definition of inflammatory markers

Based on the obtained peripheral blood cell counts, we calculated seven inflammation biomarkers: SIRI,MLR,AISI,NMLR,NLR,PLR and SII. The calculations were defined as: SIRI=neutrophil counts×monocyte counts/lymphocyte counts, MLR = monocyte counts/lymphocyte counts, AISI=neutrophil counts×platelet counts×monocyte counts/lymphocyte counts, NMLR=(monocyte counts + neutrophil counts)/lymphocyte counts, NLR=neutrophil counts/lymphocyte counts, PLR=platelet counts/lymphocyte counts, SII=platelet counts×neutrophil counts/lymphocyte counts.

### The definition of AF

In the NHANES database, according to the 10th revision of the International Classification of Diseases, the primary outcome of the study was atrial fibrillation (code I48) (23).

In the inpatient case management system of Zibo Central Hospital. All participants underwent a 12-lead ECG and were examined by a cardiologist. The diagnosis of AF is based on irregular R-R intervals, absence of regular P waves, and disordered atrial activation on ECG.

### Echocardiography

Transthoracic echocardiography was performed in all participants to record the anteroposterior diameter of the left atrium.

### The covariates

In the NHANES database, we included the following covariates: age (years), gender (male/female), race (Mexican American/non-Hispanic White/non-Hispanic Black/other races), and educational attainment classified as less than high school, high school or equivalent, and more than high school. Body Mass Index (BMI) was categorized as <18.5, 18.5–24.9, 25–29.9, and ≥30 kg/m^2^, corresponding to underweight, normal weight, overweight, and obesity, respectively. Smoking status was classified as current smokers (100 cigarettes or more in lifetime, currently smoking some days or every day) or never smokers (including those who never smoked [<100 cigarettes in lifetime] or former smokers [100 cigarettes or more in lifetime, but now never smoke]). Activity status was classified as active (engaging in moderate or vigorous work, exercise, and recreational activities) or inactive (engaging in non-moderate or non-vigorous work, exercise, and recreational activities). Hypertension was defined as (I) average systolic blood pressure ≥140 mmHg, (II) average diastolic blood pressure ≥90 mmHg, (III) current use of antihypertensive medication, or (IV) self-reported hypertension. Diabetes was defined as self-reported diagnosis of diabetes and use of diabetes medication or insulin. Hyperlipidemia was classified according to the Adult Treatment Panel III (ATP 3) of the National Cholesterol Education Program (NCEP) as total cholesterol ≥200 mg/dL, triglycerides ≥150 mg/dL, HDL <40 mg/dL in males and <50 mg/dL in females, or low-density lipoprotein ≥130 mg/dL. Alternatively, individuals who reported using cholesterol-lowering drugs were also classified as having hyperlipidemia. Coronary heart disease (CHD) was defined as self-reported coronary heart disease. Myocardial infarction (MI) was defined as self-reported myocardial infarction. In inpatient Case Management System of Zibo Central Hospital, we collected comprehensive patient data, including age, gender, hypertension, diabetes, BMI and other related indicators. Clinical examination and laboratory test results were also collected. Including lymphocyte count, monocyte count, neutrophil count, platelet count, alanine aminotransferase (ALT), aspartate aminotransferase (AST), total cholesterol (TC), triglyceride (TG), low density lipoprotein cholesterol (LDLC), high density lipoprotein cholesterol (HDLC), blood glucose level (GLU), Serum uric acid concentration, serum creatinine level.

### Statistical methods

This study used the NHANES database. Because the NHANES sampling design is complex, participant data from 2013 to 2020 were weighted according to the analysis and reporting guidelines to ensure national representativeness. Continuous variables are presented as mean ± standard deviation (SD), and categorical variables as proportions. Differences in baseline characteristics between non-AF and AF groups were assessed using weighted Student's *t*-tests for continuous variables and weighted chi-square tests for categorical variables. Inflammatory markers were categorized into tertiles based on their unweighted distributions within the NHANES cohort for analysis. First, survey-weighted multivariable logistic regression was used to assess the association between inflammatory markers and AF in three models. We first analyzed a crude model (Model 1). Next, we adjusted for age, gender, race, and education level (Model 2). Finally, we adjusted for age, gender, race, education level, BMI, smoking status, activity status, hypertension, diabetes, hyperlipidemia, myocardial infarction, and coronary heart disease (Model 3). These three multivariable logistic regression models were used to estimate odds ratios (ORs) and 95% confidence intervals (CI) for the relationship between inflammatory markers and AF. Additionally, we generated restricted cubic spline (RCS) plots to assess potential nonlinear associations between inflammatory markers and AF. We also conducted subgroup and interaction analyses across all covariates to evaluate the robustness of the findings. Similarly, we used the inpatient case management system of Zibo Central Hospital to analyze patients' baseline characteristics. Continuous variables are presented as mean ± standard deviation (SD), and categorical variables are presented as proportions. Differences in baseline characteristics between the non-AF and AF groups were compared using *t*-tests for continuous variables and weighted chi-square tests for categorical variables.We also applied survey multivariate logistic regression to assess the association between inflammatory markers and AF. First, we fitted an unadjusted model (Model I). Next, we adjusted for age, sex, BMI, hypertension, and diabetes (Model II). Finally, we additionally adjusted for ALT, AST, triglycerides, cholesterol, HDL, LDL, blood glucose, uric acid, and creatinine (Model III). These three models were used to estimate odds ratios (ORs) and 95% confidence intervals (CIs) for the associations between inflammatory markers and AF. In addition, we used mediation analysis to quantify the direct effect of SIRI on AF risk and the indirect effect mediated by atrial diameter.

Data analysis was conducted using Empower Stats (version 4.0), Stata MP 16.0 and R studio (version 4.3.0). Finally, all statistical tests were two-sided, and a *p*-value < 0.05 was considered statistically significant.

## Results

### General characteristics of the study population

Using the NHANES 2013–2020 dataset, we analyzed 10,474 participants. The cohort had a mean age of 54.21 ± 16.56 years and comprised 43.08% males and 56.92% females. Participants were classified as AF patients (*n* = 136) or non-AF patients (*n* = 10,338). Significant differences between the AF and non-AF groups were observed for age, sex, race, education level, BMI, smoking status, hypertension, hyperlipidemia, coronary heart disease, and CBC-derived indicators (all *p* < 0.05). Marital status did not differ between the groups. Compared with participants without AF, those with AF were more often older, male, predominantly non-Hispanic white, more highly educated, and had higher BMI. Additionally, the prevalence of comorbidities was higher among patients with AF, including hypertension, diabetes, myocardial infarction, and coronary heart disease (Table 1). Using the inpatient case system of Zibo Central Hospital, we identified 13,706 participants. The mean age was 63.50 ± 11.65 years; males comprised 50.67% and females 49.33% of the sample. Participants were classified into an AF group (*n* = 1722) and a non-AF group (*n* = 11,984). Most laboratory indices differed significantly between the AF and non-AF groups, with the exception of alanine aminotransferase, aspartate aminotransferase, high-density lipoprotein cholesterol, and glucose (all *p* < 0.05). In addition, patients with AF had a higher incidence of comorbidities such as hypertension and diabetes (Supplementary Table S1).

**Table 1:** Baseline characteristics of participants included in NHANES.

Variables	Overall	Non - AF	AF	*P*-value
	(*n* = 10,474)	(*n* = 10,338)	(*n* = 136)	
Age	54.21 ± 16.56	53.97 ± 16.46	71.45 ± 9.99	<0.001
Gender				0.009
Male	43.08	42.93	53.61	
Female	56.92	57.07	46.39	
Race				<0.001
Mexican American	10.71	10.81	3.96	
Non-Hispanic White	71.61	71.37	88.52	
Non-Hispanic Black	9.91	10.02	2.37	
Others	7.77	7.80	5.15	
Education level				0.013
<High school	11.45	11.55	4.56	
High school	23.72	23.63	30.04	
College or more	64.83	64.82	65.40	
Marital status				0.487
Married	60.16	60.12	62.94	
Unmarried	39.84	39.88	37.06	
BMI, kg/m^2^				0.018
<18.5	1.17	1.18	3.21	
18.5–24.9	22.10	21.06	25.99	
25–29.9	31.62	31.09	33.94	
≥30.0	45.11	46.67	36.86	
Smoking status				<0.001
Ever	26.69	26.36	49.56	
Never	53.48	53.61	44.05	
Current	19.83	20.03	6.39	
Activity status				0.135
Active activities	74.99	75.07	69.70	
Non-active activities	25.01	24.93	30.30	
Hyperlipidemia				<0.001
YES	61.08	61.35	41.89	
NO	38.92	38.65	58.11	
Diabetes				0.152
YES	18.63	18.56	23.18	
NO	81.37	81.44	76.82	
Hypertension				<0.001
YES	55.34	55.04	76.46	
NO	44.66	44.96	23.54	
Myocardial infarct				<0.001
YES	5.33	5.22	12.65	
NO	94.67	94.78	87.35	
CHD				<0.001
YES	6.19	5.99	20.41	
NO	93.81	94.01	79.59	
CBC count,10^3^/*μ*L				
Lymphocyte count	2.16 ± 3.09	2.17 ± 3.11	1.80 ± 0.64	0.156
Neutrophils count	4.41 ± 1.72	4.40 ± 1.72	4.61 ± 1.68	0.158
Monocyte count	0.60 ± 0.21	0.60 ± 0.21	0.66 ± 0.21	<0.001
Platelet count	239.74 ± 62.69	240.11 ± 62.74	213.52 ± 53.31	<0.001
CBC-derived indicators				
SIRI	1.40 ± 1.00	1.39 ± 0.99	2.00 ± 1.63	<0.001
MLR	0.31 ± 0.14	0.31 ± 0.14	0.41 ± 0.23	<0.001
AISI	339.60 ± 281.70	338.23 ± 279.45	435.87 ± 398.13	<0.001
NMLR	2.61 ± 1.39	2.60 ± 1.38	3.32 ± 1.87	<0.001
NLR	2.30 ± 1.2974	2.29 ± 1.29	2.91 ± 1.68	<0.001
PLR	124.61 ± 50.61	124.49 ± 50.40	132.93 ± 63.07	0.044
SII	549.87 ± 352.59	548.80 ± 351.50	624.71 ± 415.57	0.009

Data were *n* (%) or mean ± SD.

### Relationship between atrial fibrillation and inflammatory markers in the NHANES

In Model 1, all the inflammation markers derived from complete blood cell count, namely SIRI, MLR, AISI,NMLR, NLR, PLR and SII, were positively correlated with AF, with OR values of 1.334 (95% CI: 1.199, 1.282; *P* < 0.001), 14.621 (95% CI: 6.802, 31.430; *P* < 0.001), 1.001 (95% CI: 1.000, 1.001; *P* = 0.001), 1.200 (95% CI: 1.124, 1.283; *P* < 0.001), 1.198 (95% CI: 1.118, 1.283; *P* < 0.001), 1.003 (95% CI: 0.999, 1.006; *P* < 0.001), and 1.000 (95% CI: 1.000, 1.001; *P* = 0.014), respectively. In Model 2 and 3, the correlations between SIRI and MLR and AF remained stable, with OR values of 1.158 (95% CI: 1.024, 1.310; *P* = 0.020), 1.147 (95% CI: 1.017, 1.294; *P* = 0.026) and 3.060 (95% CI: 1.258, 7.441; *P* = 0.014), 2.775 (95% CI: 1.163, 6.618; *P* = 0.021), respectively. However, in Model 2 and 3, AISI was not significantly correlated with AF, with OR values of 1.000 (95% CI: 0.999, 1.001; *P* = 0.175), 1.000 (95% CI: 1.000, 1.001; Moreover, the association results of NMLR, NLR, PLR, and SII were similar to AISI showing no significant correlation with AF (*P* > 0.05).When inflammation markers were categorized by tertiles, Model 1 showed that the middle SIRI tertile (Q2) and highest SIRI tertiles (Q3) had greater odds of AF than the lowest tertiles (Q1), with ORs of 2.072 (95% CI: 1.064, 4.033; *P* = 0.032) and 4.673 (95% CI: 2.548, 8.572; *P* < 0.001), respectively. In Models 2 and 3, the highest SIRI tertiles (Q3) remained positively associated with AF vs. Q1, with an OR of 2.345 (95% CI: 1.208, 4.554; *P* = 0.012). In Model 1, compared with the lowest MLR tertile (Q1), the middle MLR tertiles (Q2) and the highest MLR tertiles (Q3) were positively associated with AF, with ORs of 2.277 (95% CI: 1.040, 4.983; *P* = 0.039) and 5.477 (95% CI: 2.854, 10.509; *P* < 0.001), respectively. However, In Model 2 and Model 3, neither the middle MLR tertiles (Q2) nor the highest MLR tertiles (Q3) showed a significant association with AF (*P* > 0.05). The association patterns for AISI and MLR mirrored those observed for MLR. In Model 1, compared with the lowest tertile (Q1), the highest NLR tertiles (Q3) was positively associated with AF, with an OR of 3.165 (95% CI: 1.708, 5.87; *P* < 0.001). However, in Models 2 and 3, neither the middle NLR tertiles (Q2) nor the highest NLR tertiles (Q3) differed significantly from the lowest NLR tertiles (Q1) with respect to AF. The ORs were 1.200 (95% CI: 0.621, 2.321; *P* = 0.587), 1.138 (95% CI: 0.582, 2.227; *P* = 0.706), 1.615 (95% CI: 0.847, 3.076; *P* = 0.145), and 1.507 (95% CI: 0.787, 2.883; *P* = 0.216), respectively. The three NLR tertiles produced association results similar to those of NMLR. Across Models 1, 2, and 3, none of the three tertiles of PLR or SII was significantly associated with atrial fibrillation (*P* > 0.05) (Table 2).

**Table 2:** Logistic regression analysis on atrial fibrillation in NHANES.

Characteristics	Model1		Model2		Model3	
	OR (95% CI)	*P*-Value	OR (95% CI)	*P*-Value	OR (95% CI)	*P*-Value
SIRI (continuous)	1.334 (1.199,1.484)	<0.001	1.158 (1.024,1.310)	0.020	1.147 (1.017,1.294)	0.026
SIRI (tertiles)						
Q1 (<0.860)	reference		reference		reference	
Q2 (0.860∼1.429)	2.072 (1.064,4.033)	0.032	1.417 (0.712,2.818)	0.321	1.367 (0.680,2.747)	0.380
Q3 (>1.429)	4.673 (2.548,8.572)	<0.001	2.345 (1.208,4.554)	0.012	2.182 (1.094,4.354)	0.027
P for trend	<0.001		<0.001		<0.001	
MLR (continuous)	14.621 (6.802,31.430)	<0.001	3.060 (1.258,7.441)	0.014	2.775 (1.163,6.618)	0.021
MLR (tertiles)						
Q1 (<0.231)	reference		reference		reference	
Q2 (0.231∼0.320)	2.277 (1.040,4.983)	0.039	1.506 (0.673,3.368)	0.319	1.495 (0.676,3.304)	0.321
Q3 (>0.320)	5.477 (2.854,10.509)	<0.001	2.004 (0.970,4.138)	0.060	1.933 (0.936,3.991)	0.075
P for trend	<0.001		<0.001		<0.001	
AISI (continuous)	1.001 (1.000,1.001)	0.001	1.000 (0.999,1.001)	0.175	1.000 (1.000,1.001)	0.137
AISI(tertiles)						
Q1 (<191.546)	reference		reference		reference	
Q2 (191.546∼340.979)	1.963 (1.078,3.570)	0.027	1.567 (0.839,2.924)	0.158	1.563 (0.828,2.95)	0.168
Q3 (>340.979)	2.409 (1.407,4.123)	0.001	1.564 (0.892,2.742)	0.118	1.523 (0.838,2.768)	0.168
P for trend	<0.001		<0.001		<0.001	
NMLR (continuous)	1.200 (1.124,1.283)	<0.001	1.082 (0.998,1.173)	0.057	1.077 (0.992,1.169)	0.076
NMLR (tertiles)						
Q1 (<1.871)	reference		reference		reference	
Q2 (1.871∼2.706)	1.610 (0.836,3.100)	0.155	1.200 (0.621,2.321)	0.587	1.138 (0.582,2.227)	0.706
Q3 (>2.706)	3.165 (1.708,5.87)	<0.001	1.615 (0.847,3.076)	0.145	1.507 (0.787,2.883)	0.216
P for trend	<0.001		<0.001		<0.001	
NLR (continuous)	1.198 (1.118,1.283)	<0.001	1.080 (0.991,1.176)	0.077	1.076 (0.986,1.174)	0.099
NLR (tertiles)						
Q1 (<1.625)	reference		reference		reference	
Q2 (1.625∼2.385)	1.545 (0.801,2.981)	0.195	1.191 (0.613,2.315)	0.606	1.141 (0.591,2.201)	0.694
Q3 (>2.385)	2.851 (1.505,5.404)	0.001	1.530 (0.785,2.981)	0.212	1.427 (0.738,2.757)	0.290
P for trend	<0.001		<0.001		<0.001	
PLR (continuous)	1.003 (0.999,1.006)	<0.001	1.000 (0.997,1.004)	0.877	1.001 (0.997,1.00)	0.755
PLR (tertiles)						
Q1 (<97.836)	reference		reference		reference	
Q2 (97.836∼132.778)	1.162 (0.671,2.014)	0.593	1.152 (1.152,2.041)	0.628	1.213 (0.689,2.135)	0.504
Q3 (>132.778)	1.180 (0.714,1.948)	0.519	0.876 (0.527,1.457)	0.611	0.938 (0.562,1.566)	0.806
P for trend	<0.001		<0.001		<0.001	
SII (continuous)	1.000 (1.000,1.001)	0.014	1.000 (0.999,1.000)	0.464	1.000 (1.000,1.001)	0.407
SII (tertiles)						
Q1 (<368.412)	reference		reference		reference	
Q2 (368.412∼569.625)	0.894 (0.509,1.571)	0.697	0.803 (0.454,1.420)	0.451	0.804 (0.441,1.466)	0.476
Q3 (>569.625)	1.545 (0.922,2.588)	0.099	1.200 (0.705,2.043)	0.502	1.205 (0.693,2.095)	0.508
P for trend	<0.001		<0.001		<0.001	

OR Odds Ratio, CI Confidence Interval.

Model 1 was not adjusted for any confounders.

Model 2 was adjusted for gender, age, race and education level.

Model 3 was adjusted for gender, age, race,education level, BMI, activity status, smoke status, hyperlipidemia, diabetes, hypertension, myocardial infarct, CHD.

### Nonlinearity analysis using RCS

**Figure 2:**
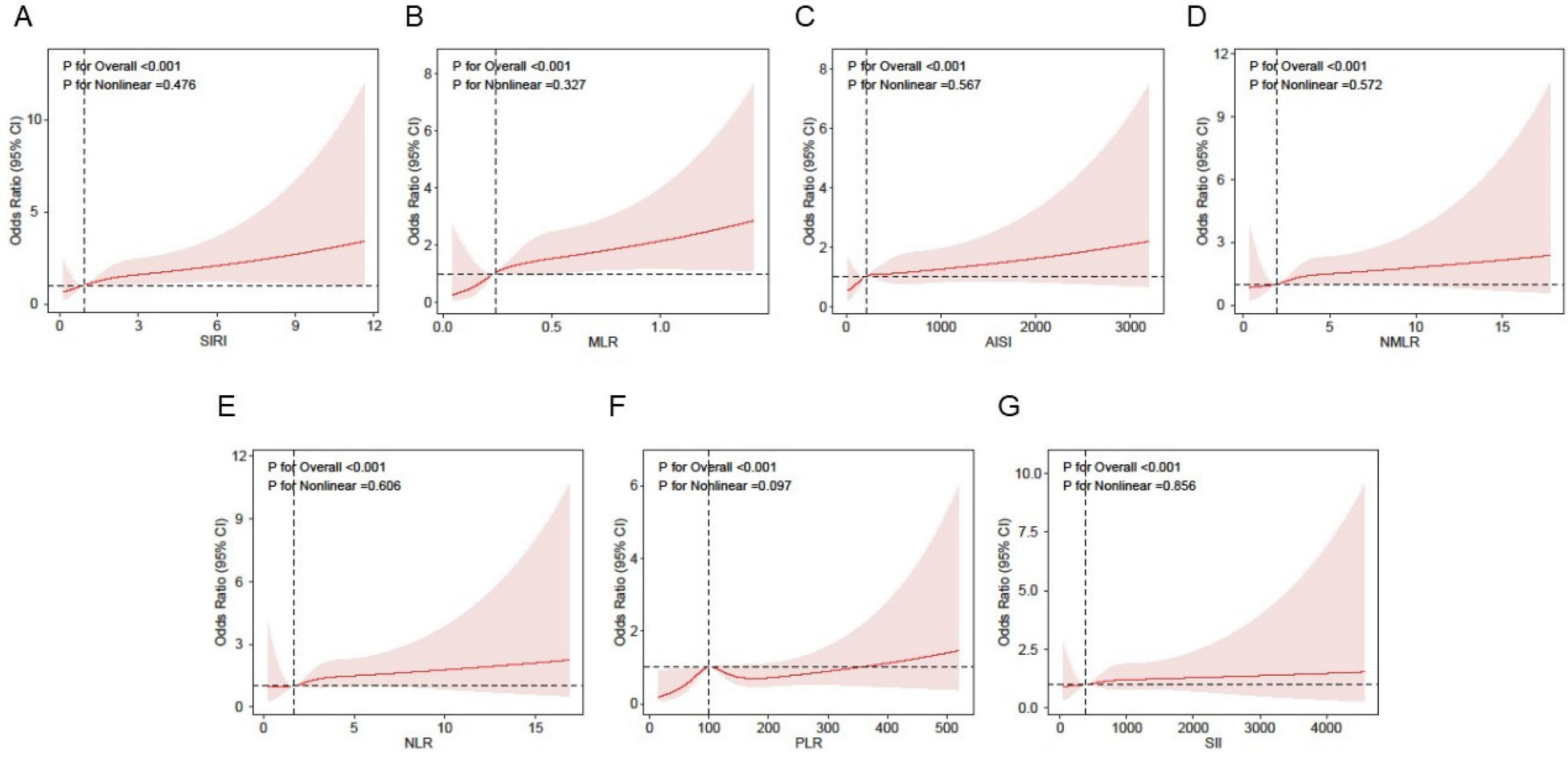
Association between inflammatory markers and AF using a restricted cubic spline regression model in NHANES. Adjusted for gender, age, race,education level, BMI, activity status, smoking status, hyperlipidemia, diabetes, hypertension, myocardial infarct and CHD. **(A)** Association of SIRI with AF risk. **(B)** Association of MLR with AF risk. **(C)** Association of AISI with AF risk. **(D)** Association of NMLR with AF risk. **(E)** Association of NLR with AF risk. **(F)** Association of PLR with depression risk. **(G)** Association of SII with AF risk. SIRI, systemic inflammation response index; MLR, monocyte-to-lymphocyte ratio; AISI, aggregate index of systemic inflammation; NMLR, neutrophil-monocyte-to-lymphocyte ratio; NLR, neutrophil-to-lymphocyte ratio PLR, platelet-to-lymphocyte ratio; SII, systemic inflammatory index.

Figure 2 presents RCS curves that further depict the association between inflammatory markers and atrial fibrillation. After adjusting for gender, age, race, education level, BMI, smoking status, activity status, hypertension, diabetes, hyperlipidemia, coronary heart disease, and myocardial infarction, the adjusted results show a significant positive association between inflammatory markers and atrial fibrillation, with a linear increasing trend (*P* < 0.001;P for non-linear *P* > 0.05).

### The subgroup analysis and interaction test

To assess the robustness of the positive association between inflammatory markers and atrial fibrillation, we performed subgroup analyses by age (categorized), gender (categorized), race (Mexican American/Non-Hispanic White/Non-Hispanic Black/Other), BMI (categorized), hypertension (No, Yes), diabetes (No, Yes), and coronary heart disease (No, Yes). All covariates were included in each subgroup model except the stratification variable. We observed no significant interactions between inflammatory markers and any of these potential confounders (all *P*-value for interaction > 0.05) (Table 3).

**Table 3:** Subgroup analysis for the association between inflammatory markers and AF.

Characteristics	SIRI		MLR		AISI		NMLR	
	OR(95%CI)	*P*-value	OR(95%CI)	*P*-value	OR(95%CI)	*P*-value	OR(95%CI)	*P*-value
Gender, *n* (%)								
Male	1.19 (1.07,1.32)	<0.001	4.76 (2.13,10.61)	<0.001	1.00 (1.00,1.00)	0.041	1.16 (1.07,1.26)	<0.001
Female	1.25 (1.11,1.42)	<0.001	10.72 (3.99,28.83)	<0.001	1.00 (1.00,1.00)	0.015	1.13 (1.02,1.24)	0.016
P-interaction	0.642		0.343		0.605		0.521	
Age, *n* (%)								
<65	1.22 (1.10,1.34)	<0.001	4.83 (2.40,9.73)	<0.001	1.00 (1.00,1.00)	0.007	1.14 (1.06,1.22)	<0.001
≥65	0.95 (0.60,1.50)	0.828	1.82 (0.12,26.66)	0.661	1.00 (1.00,1.00)	0.534	0.96 (0.68,1.37)	0.831
P-interaction	0.372		0.809		0.302		0.458	
Race, *n* (%)								
Mexican American	1.34 (1.02,1.74)	0.033	156.99 (11.24,2193.13)	<0.001	1.00 (1.00,1.00)	0.335	1.20 (0.94,1.53)	0.146
Non-Hispanic White	1.19 (1.08,1.31)	<0.001	4.69 (2.27,9.71)	<0.001	1.00 (1.00,1.00)	0.053	1.13 (1.05,1.22)	<0.001
Non-Hispanic Black	1.09 (0.86,1.40)	0.475	4.69 (2.27,9.71)	0.487	1.00 (1.00,1.00)	0.370	1.02 (0.75,1.37)	0.920
Others	1.00 (0.45,2.20)	0.997	16.51 (0.07,4095.58)	0.319	1.00 (0.99,1.00)	0.445	0.81 (0.28,2.33)	0.694
P-interaction	0.830		0.110		0.833		0.777	
BMI, *n* (%)								
<18.5	0.53 (0.03,10.34)	0.679	7.94 (0.00,100489.56)	0.667	1.00 (0.98,1.01)	0.562	0.94 (0.17,5.30)	0.948
18.5–24.9	1.13 (0.98,1.31)	0.099	4.13 (0.84,20.43)	0.082	1.00 (1.00,1.00)	0.257	1.09 (0.94,1.28)	0.251
25–29.9	1.22 (1.08,1.39)	0.002	4.73 (1.97,11.31)	<0.001	1.00 (1.00,1.00)	0.015	1.12 (1.03,1.23)	0.009
≥30.0	1.31 (1.15,1.50)	<0.001	16.97 (5.98,48.15)	<0.001	1.00 (1.00,1.00)	0.132	1.23 (1.11,1.36)	<0.001
P-interaction	0.264		0.345		0.439		0.306	
Diabetes, *n* (%)								
NO	1.20 (1.09,1.31)	<0.001	8.14 (4.00,16.56)	<0.001	1.00 (1.00,1.00)	0.033	1.14 (1.07,1.23)	<0.001
YES	1.27 (1.09,1.48)	0.002	4.92 (1.50,16.13)	0.008	1.00 (1.00,1.00)	0.053	1.17 (1.03,1.33)	0.017
P-interaction	0.572		0.266		0.848		0.899	
Hypertension, *n* (%)								
NO	1.16 (1.00,1.35)	0.053	5.26 (1.64,16.86)	0.005	1.00 (1.00,1.00)	0.297	1.10 (0.98,1.24)	0.117
YES	1.22 (1.11,1.33)	<0.001	6.74 (3.26,13.94)	<0.001	1.00 (1.00,1.00)	0.011	1.16 (1.08,1.25)	<0.001
P-interaction	0.920		0.919		0.965		0.742	
CHD, *n* (%)								
NO	1.22 (1.12,1.32)	<0.001	7.30 (3.79,14.06)	<0.001	1.00 (1.00,1.00)	0.005	1.17 (1.09,1.25)	<0.001
YES	1.18 (0.95,1.47)	0.140	3.48 (0.55,22.02)	0.186	1.00 (1.00,1.00)	0.285	1.04 (0.87,1.24)	0.675
P-interaction	0.665		0.339		0.984		0.112	

Characteristics	NLR		PLR		SII	
	OR(95%CI)	*P*-value	OR(95%CI)	*P*-value	OR(95%CI)	*P*-value
Gender, *n* (%)						
Male	1.17 (1.07,1.28)	<0.001	1.00 (1.00,1.01)	0.264	1.00 (1.00,1.00)	0.052
Female	1.12 (1.01,1.25)	0.038	1.00 (1.00,1.00)	0.955	1.00 (1.00,1.00)	0.542
P-interaction	0.470		0.416		0.455	
Age, *n* (%)						
<65	1.14 (1.05,1.23)	0.001	1.00 (1.00,1.00)	0.583	1.00 (1.00,1.00)	0.063
≥65	0.95 (0.64,1.40)	0.784	1.00 (0.99,1.01)	0.450	1.00 (1.00,1.00)	0.355
P-interaction	0.445		0.365		0.175	
Race, *n* (%)						
Mexican American	1.18 (0.89,1.57)	0.236	1.00 (0.99,1.01)	0.749	1.00 (1.00,1.00)	0.925
Non-Hispanic White	1.14 (1.05,1.23)	0.002	1.00 (1.00,1.00)	0.719	1.00 (1.00,1.00)	0.208
Non-Hispanic Black	1.01 (0.71,1.43)	0.975	1.00 (0.99,1.01)	0.914	1.00 (1.00,1.00)	0.801
Others	0.71 (0.20,2.50)	0.59	0.97 (0.94,1.01)	0.170	1.00 (0.99,1.00)	0.148
P-interaction	0.776		0.324		0.408	
BMI, *n* (%)						
<18.5	0.86 (0.12,6.19)	0.885	1.03 (0.99,1.08)	0.180	1.00 (0.99,1.01)	0.720
18.5–24.9	1.10 (0.92,1.30)	0.291	1.00 (0.99,1.01)	0.543	1.00 (1.00,1.00)	0.770
25–29.9	1.13 (1.02,1.24)	0.015	1.00 (1.00,1.01)	0.158	1.00 (1.00,1.00)	0.079
≥30.0	1.23 (1.10,1.37)	<0.001	1.00 (1.00,1.00)	0.992	1.00 (1.00,1.00)	0.342
P-interaction	0.315		0.517		0.503	
Diabetes, *n* (%)						
NO	1.15 (1.06,1.23)	<0.001	1.00 (1.00,1.00)	0.525	1.00 (1.00,1.00)	0.241
YES	1.18 (1.02,1.35)	0.024	1.00 (1.00,1.01)	0.732	1.00 (1.00,1.00)	0.125
P-interaction	0.878		0.873		0.532	
Hypertension, *n* (%)						
NO	1.10 (0.96,1.26)	0.160	1.00 (1.00,1.01)	0.770	1.00 (1.00,1.00)	0.640
YES	1.16 (1.07,1.26)	<0.001	1.00 (1.00,1.00)	0.569	1.00 (1.00,1.00)	0.098
P-interaction	0.758		0.844		0.819	
CHD, *n* (%)						
NO	1.17 (1.09,1.26)	<0.001	1.00 (1.00,1.00)	0.217	1.00 (1.00,1.00)	0.036
YES	1.03 (0.85,1.25)	0.752	1.00 (0.99,1.00)	0.620	1.00 (1.00,1.00)	0.992
P-interaction	0.114		0.233		0.355	

The model was adjusted for gender(categorical),age (categorical), race (Mexican American/non-Hispanic White/non-Hispanic Black/other races), BMI (categorical), Hypertension(No, Yes),Diabetes (No, Yes), and CHD(No, Yes). All covariates in the subgroup analysis models were adjusted, excepting the stratification variable itself.

### Relationship between AF and inflammation markers

Our results show a significant association between higher SIRI and greater odds of AF. Model I reported an OR of 1.112 (95% CI 1.070,1.156; *P* < 0.001). Model II likewise showed a significant association (OR: 1.088; 95% CI 1.045,1.132; *P* < 0.001). In Model III the association persisted (OR: 1.081; 95% CI 1.038,1.127; *P* < 0.001). In a sensitivity analysis treating SIRI as a categorical variable, participants in the highest tertile had a 43.6% increased risk of AF compared with those in the lowest tertile (OR: 1.436; 95% CI 1.248,1.652; *P* < 0.001) (Table 4).

**Table 4:** Logistic regression analysis on atrial fibrillation.

Characteristics	Model Ⅰ		Model Ⅱ		Model Ⅲ	
	OR (95% CI)	*P*-Value	OR (95% CI)	*P*-Value	OR (95% CI)	*P*-Value
SIRI (continuous)	1.112 (1.070,1.156)	<0.001	1.088 (1.045,1.132)	<0.001	1.081 (1.038,1.127)	<0.001
SIRI (tertiles)						
Q1 (<0.653)	reference		reference		reference	
Q2 (0.653∼1.063)	1.807 (1.586,2.058)	<0.001	1.663 (1.456,1.899)	<0.001	1.664 (1.456,1.902)	<0.001
Q3 (>1.063)	1.656 (1.451,1.890)	<0.001	1.451 (1.263,1.666)	<0.001	1.436 (1.248,1.652)	<0.001
P for trend	<0.001		<0.001		<0.001	

OR, odds ratio; CI, confidence interval.

Model Ⅰ was not adjusted for any confounders.

Model Ⅱ was adjusted for gender, age, BMI, hypertension and diabetes.

Model Ⅲ was adjusted for gender, age, BMI, hypertension, diabetes, ALT, AST, TC, TG, LDLC, HDLC, GLU, Serum uric acid concentration, serum creatinine level.

### Mediation analysis

**Figure 3:**
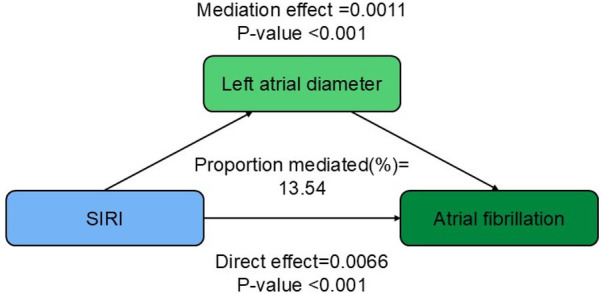
Path diagram of the mediation analysis of left atrial diameter on the relationship between SIRI and AF.

The mediation analysis shown in Figure 3 indicates that an increase in SIRI is consistently associated with a higher risk of AF, even after adjusting for covariates. The results further indicate that part of this association is mediated by left atrial diameter, with a mediation proportion of 13.54% (*P* < 0.001).

## Discussion

This cross-sectional study included 10,474 eligible participants from NHANES and 13,706 eligible participants from Zibo Central Hospital. We examined the association between inflammatory markers derived from complete blood cell counts and AF. After adjusting for multiple covariates, SIRI showed a statistically significant positive association with AF, indicating that higher SIRI correlates with greater AF risk. Restricted cubic spline analysis revealed a linear dose–response relationship between these inflammatory markers and AF risk. Interaction tests and subgroup analyses stratified by selected clinical parameters and comorbidities found no significant interactions. Notably, left atrial diameter partially mediated the association between SIRI and AF.

Inflammation is closely linked to the onset and persistence of atrial fibrillation. Inflammatory mediators produced by mononuclear macrophages, such as TNF-α and IL-6, promote atrial myocyte degeneration and fibrosis and slow conduction, thereby contributing to both structural and electrical remodeling that can initiate and sustain atrial fibrillation (4, 7). Conversely, in patients with atrial fibrillation the renin–angiotensin–aldosterone system (RAAS) is activated because of reduced cardiac function and hypertension (24). Ang II enhances neutrophil recruitment and stimulates secretion of inflammatory cytokines including IL-6, IL-8, and TNF-α (25). This establishes a vicious cycle in which left atrial diameter, a key structural marker, is tightly associated with inflammation and with atrial fibrillation. Atrial fibrillation produces abnormal atrial electrical activity that impairs systolic and diastolic function and, over time, leads to atrial enlargement; left atrial enlargement in turn is an important risk factor for atrial fibrillation. Notably, plasma concentrations of inflammatory markers such as IL-6 correlate positively with LA diameter (12, 13), indicating that inflammation may exacerbate AF progression by promoting atrial structural remodeling, particularly left atrial enlargement. In recent years, inflammatory indices derived from whole blood cells (e.g., SIRI, MLR, AISI, NMLR, NLR, PLR, and SII) have emerged as robust biomarkers across diverse diseases (26–31). Among these, the systemic inflammation response index (SIRI) outperforms many conventional markers in predicting disease progression and evaluating clinical outcomes, a finding supported by multicenter data. In patients with coronary heart disease, SIRI correlates strongly with disease severity (32, 33). Studies across varied populations indicate that SIRI can stratify atrial fibrillation risk and inform individualized management, offering a practical tool for early identification of high-risk individuals (21, 34). Other work links SIRI to cardiovascular and all-cause mortality, underscoring the role of systemic inflammation in prevention strategies (20). Finally, in acute coronary syndrome, higher post–percutaneous coronary intervention SIRI predicts increased risk of major adverse cardiovascular events (MACE) (26, 35).

In the baseline characteristics, patients with atrial fibrillation had a significantly higher mean body mass index (BMI) than those without atrial fibrillation, indicating that obesity may be an important risk factor for the onset and progression of atrial fibrillation. This finding aligns with extensive epidemiological evidence showing that obesity is independently associated with the incidence and prevalence of atrial fibrillation (36). Epicardial adipose tissue (EAT), a specialized fat depot located between the myocardial surface and the visceral pericardium, has recently emerged as a key mediator linking obesity to atrial fibrillation, particularly by promoting atrial remodeling through inflammatory mechanisms. In obesity, EAT volume increases markedly, and the inflammatory mediators it secretes can drive atrial structural remodeling by multiple pathways. Systemically, obesity provokes a proinflammatory state that amplifies EAT inflammatory activity, leading to left atrial fibrosis and chamber enlargement and thereby facilitating atrial fibrillation (37). Local changes also contribute: in human and sheep models of atrial fibrillation, subepicardial adipose tissue shows thickening and fibrotic infiltration (38); these changes reinforce one another and create a vicious cycle. Research indicates that patients with elevated SIRI have significantly greater volumes of EAT (39, 40). Moreover, EAT mediates the relationship between SIRI and myocardial fibrosis, implying that systemic inflammation can exacerbate myocardial fibrosis indirectly by altering EAT function. Goette et al. identified left atrial fibrosis and enlargement of the inner diameter as core structural remodeling features of ACM that directly increase AF risk (41). Pierucci et al. elucidated the inflammatory mechanisms associated with ACM and highlighted the pro-inflammatory role of EAT. EAT releases pro-inflammatory factors that facilitate local inflammatory infiltration and atrial fibrosis, thereby contributing to the onset and persistence of atrial fibrillation (42).

Atrial structural and electrical remodeling constitute key pathophysiological substrates for AF. Atrial fibrosis, a hallmark of structural remodeling, disrupts local electrical coupling, facilitates reentrant circuits and regional conduction block, and thereby creates a substrate for AF. The heart's dense autonomic innervation—sympathetic, parasympathetic, and intrinsic cardiac ganglia—exerts a major influence on atrial electrophysiology and arrhythmogenesis. Interventions that reduce autonomic innervation or activation lower the incidence of atrial arrhythmias, indicating that autonomic modulation can help prevent AF (43). Clinically, atrial enlargement predicts AF development. Cardiac magnetic resonance, speckle-tracking echocardiography, electroanatomic voltage mapping, and circulating biomarkers permit quantitative assessment of myocardial fibrosis and inform treatment decisions and recurrence risk in patients with AF. Underlying heart diseases (such as hypertension, valvular heart disease, and heart failure) can exacerbate atrial fibrosis through multiple mechanisms. Chronic pressure or volume overload raises atrial wall tension and activates mechanosensitive signaling pathways, which upregulate pro-fibrotic gene expression. Activation of neuroendocrine systems, such as the renin-angiotensin-aldosterone system, further promotes inflammation and fibrotic remodeling.

This study indicates that the association between SIRI and AF risk is partly mediated by left atrial diameter, reinforcing the link between inflammation and atrial remodeling in atrial fibrillation pathogenesis. This discovery holds potential implications for clinical practice, especially for the selection of ablation treatment strategies for atrial fibrillation. For assessing the level of systemic inflammation, it may help identify those patient groups with more active atrial remodeling, who may face a higher risk of recurrence after ablation due to persistent structural and electrical remodeling. Prior work shows that pre-ablation SII predicts recurrence after cryoballoon ablation (44), and that an increased NLR on day 1 after radiofrequency catheter ablation independently predicts early recurrence (≤3 months) in atrial fibrillation patients (45). Different ablation modalities also appear to differentially affect the inflammatory milieu; for example, radiofrequency catheter ablation may elicit greater inflammation than cryoballoon ablation (46). Future studies should test whether inflammatory markers can guide individualized selection of ablation technique or timing to improve procedural success and reduce recurrence.

Several anti-inflammatory strategies show promise in cardiovascular disease. Colchicine, a recognized anti-inflammatory agent, has demonstrated mechanistic effects in atrial fibrillation in animal studies (47), including inhibition of IL-6 release triggered by IL-1β, attenuation of subsequent atrial fibrosis, and modulation of ion channel gene expression and signaling pathways. These findings indicate potential utility for inflammation-driven arrhythmias. Corticosteroids have been used in inflammatory cardiomyopathy despite dose-limiting side effects, and targeted study of an AF subpopulation with clear systemic inflammation may be warranted (48). More selective approaches include biological agents such as IL-6 inhibitors, which are approved for autoimmune diseases. Current evidence shows that tocilizumab significantly reduces new-onset atrial fibrillation in patients with severe COVID-19 (49). Given IL-6's role in atrial remodeling and its association with left atrial enlargement, IL-6 blockade may offer a novel strategy to diminish atrial inflammatory matrix and slow progression of atrial fibrillation.

This study is the first to comprehensively evaluate the association between whole blood count–derived inflammatory markers and atrial fibrillation (AF) using large-scale population data from NHANES and inpatient records from Zibo Central Hospital, and to validate SIRI as a novel, readily available inflammatory predictor of AF. The left atrial diameter partially mediates the relationship between SIRI and AF risk. Although the data lack information on specific AF etiologies, which may account for some heterogeneity of results across AF subtypes, we consistently observed lower lymphocyte counts and higher SIRI values in patients with AF compared with non-AF patients, suggesting a link between AF onset and systemic inflammation. These findings introduce additional inflammatory metrics for AF risk stratification. We recommend incorporating CBC-derived inflammatory markers, particularly SIRI, into AF risk assessment frameworks to improve monitoring of inflammatory status and support earlier intervention in high-risk individuals.

This study is the first to suggest SIRI as a novel and readily available inflammatory marker that is significantly associated with AF risk in large population data, with left atrial diameter acting as a partial mediator. These findings provide a new inflammatory marker for risk stratification of AF; It provides a basis for screening and early intervention of AF high-risk population. Although this study had a cross-sectional design and causality cannot be inferred, the results provide an important foundation for future prospective intervention studies. We suggest that CBC-derived inflammatory markers, especially SIRI, should be included in the AF risk assessment system, and the monitoring and management of inflammatory states should be strengthened in clinical practice. This study has several important limitations. First, the cross-sectional design precludes the determination of causal directions, necessitating consideration of the potential for reverse causality. The observed elevation in inflammatory markers may indicate a consequence of atrial fibrillation rather than its etiology, or there may exist a mutually reinforcing cycle between the two. Future research should focus on elucidating causal sequences through longitudinal cohort studies or interventional experimental designs. Second, AF was ascertained exclusively by ICD codes and self-report, which may introduce misclassification bias when asymptomatic or paroxysmal cases are common. Third, inflammatory markers were measured once, so intra-individual variability is not captured and the uncertainty in association estimates is increased. Fourth, although multiple covariates were adjusted for residual confounding remains possible. The dataset lacks some potentially important confounders, including specific use of statins, corticosteroids, nonsteroidal anti-inflammatory drugs, and anticoagulants, which can directly influence inflammation and atrial fibrillation risk. In addition, NHANES does not include canonical inflammatory markers such as IL-6 and TNF-α, limiting deeper exploration of inflammatory pathways. This study used the anteroposterior diameter of the left atrium (LAD) to represent left atrial size and examined its potential mediating role between systemic inflammation and atrial fibrillation. Although left atrial volume (LAV) is considered a more comprehensive and accurate measure of left atrial structure, we selected left atrial diameter (LAD) in this study for the following reasons. First, in routine echocardiographic practice, LAD is more widely available and standardized. Second, prior studies have established an association between LAD and atrial fibrillation risk. We acknowledge, however, that LAD as a linear metric may not fully capture geometric remodeling or functional change of the left atrium. Incorporating three-dimensional measures such as LAV in future studies would better delineate the pathological link between inflammation and atrial structural changes. Finally, our hospital dataset derives from a retrospective clinical diagnosis and treatment database. In routine practice, particularly when screening and managing large, heterogeneous inpatient populations, the scope of cardiac ultrasound is often constrained by clinical indications, available resources, and documentation practices. The database primarily contains basic echocardiographic parameters, such as the left atrial anteroposterior diameter (LAD) used in this study. More refined measures—left atrial volume index (LAVI), tissue Doppler–derived diastolic indices (e.g., E/e'), estimated pulmonary artery systolic pressure (PASP), and left ventricular global longitudinal strain (GLS)—which offer greater pathophysiological discrimination, are recorded far less frequently in our dataset. As a result, large-scale, statistically robust analyses of these variables are not feasible. In the NHANES and Zibo Central Hospital cohorts, we adjusted for multiple cardiovascular risk factors and comorbidities. However, the extremely small number of young atrial fibrillation cases precluded a subgroup analysis focused on the young population. Consequently, this study cannot provide specific risk estimates for that subgroup. These limitations indicate that our conclusions require confirmation in more rigorous prospective studies that incorporate precise electrocardiographic diagnosis, a panel of biomarkers, and more comprehensive medication data. Overall, this study supplies population-based cross-sectional evidence supporting the inflammatory hypothesis of atrial fibrillation and establishes a hypothesis-generating foundation for future mechanistic and clinical research.

## Conclusion

The results showed that SIRI was significantly and positively associated with increased risk of AF, and further mediation analysis suggested that left atrial diameter enlargement was a potential mediating pathway in this association. However, it is necessary to further explore the causal relationship between these inflammatory markers and AF in the future.

## Data Availability

The original contributions presented in the study are included in the article/Supplementary Material, further inquiries can be directed to the corresponding authors.
