# Supplementary material for: Association and impact of inflammatory markers and cardiac structure on atrial fibrillation risk: a study integrating NHANES with real-world data
Source: Front Cardiovasc Med. 2026 Jan 13;12:1724217. doi: 10.3389/fcvm.2025.1724217 (PMC12834713; doi:10.3389/fcvm.2025.1724217)
Supplement: Supplementary file 1 [file Table1.docx]

Supplementary table 1 : describes some features of patients with AF.

| **Variables** | **Overall** | **Non- AF** | **AF** | **P value** |
| --- | --- | --- | --- | --- |
|  | **n = 13706** | **n = 11984** | **n = 1722** |  |
| Age | 63.50 ± 11.65 | 63.26 ± 11.64 | 65.22 ± 11.57 | <0.001 |
| GENDER(%) |  |  |  | <0.001 |
| male | 6945 (50.67) | 5955 (49.69) | 990 (57.49) |  |
| female | 6761 (49.33) | 6029 (50.31) | 732 (42.51) |  |
| BMI | 26.03 ± 9.96 | 25.972 ± 9.52 | 26.44 ± 12.59 | 0.067 |
| BMIQ4(%) |  |  |  | 0.127 |
| <18.5 | 229 (1.67) | 206 (1.72) | 23 (1.34) |  |
| 18.5-24.9 | 5604 (40.89) | 4937 (41.20) | 667 (38.73) |  |
| 25-29.9 | 6345 (46.29) | 5515 (46.02) | 830 (48.20) |  |
| ≥30.0 | 1528 (11.15) | 1326 (11.07) | 202 (11.73) |  |
| Neutrophil count | 3.56 ± 1.40 | 3.53 ± 1.40 | 3.73 ± 1.39 | <0.001 |
| Lymphocyte count | 1.90 ± 0.65 | 1.91 ± 0.66 | 1.82 ± 0.59 | <0.001 |
| Monocyte count | 0.49 ± 0.16 | 0.48 ± 0.16 | 0.51 ± 0.17 | <0.001 |
| Platelet count | 217.45 ± 57.34 | 218.12 ± 57.14 | 212.83 ± 58.49 | <0.001 |
| Red blood cell count | 4.42 ± 0.55 | 4.41 ± 0.55 | 4.49 ± 0.56 | <0.001 |
| Hemoglobin | 134.15 ± 16.95 | 133.89 ± 16.89 | 135.89 ± 17.32 | <0.001 |
| Alanine amino transferase | 22.44 ± 43.21 | 22.36 ± 44.18 | 23.03 ± 35.76 | 0.551 |
| Aspartate amino transferase | 22.47 ± 47.27 | 22.42 ± 49.31 | 22.84 ± 29.40 | 0.733 |
| Total cholesterol | 4.31 ± 1.12 | 4.32 ± 1.12 | 4.24 ± 1.15 | 0.009 |
| Triglyceride | 1.50 ± 1.09 | 1.51 ± 1.11 | 1.42 ± 0.95 | <0.001 |
| High density lipoprotein cholesterol | 1.16 ± 0.29 | 1.16 ± 0.29 | 1.17 ± 0.29 | 0.476 |
| Low density lipoprotein cholesterol | 2.68 ± 0.91 | 2.69 ± 0.91 | 2.64 ± 0.94 | 0.034 |
| Glucose | 5.83 ± 1.77 | 5.83 ± 1.80 | 5.80 ± 1.60 | 0.557 |
| Creatinine | 70.64 ± 36.35 | 69.99 ± 34.91 | 75.18 ± 44.87 | <0.001 |
| Uric acid | 322.00 ± 93.21 | 320.29 ± 92.55 | 333.91 ± 96.87 | <0.001 |
| Atrial diameter | 34.75 ± 5.68 | 34.42 ± 5.35 | 37.07 ± 7.16 | <0.001 |
| Diabetes(%) |  |  |  | <0.001 |
| NO | 11704 (85.39) | 10308 (86.01) | 1396 (81.07) |  |
| YES | 2002 (14.61) | 1676 (13.99) | 326 (18.93) |  |
| Hypertension(%) |  |  |  | <0.001 |
| NO | 9359 (68.28) | 8391 (70.02) | 968 (56.21) |  |
| YES | 4347 (31.72) | 3593 (29.98) | 754 (43.79) |  |
| SIRI | 1.07 ± 1.05 | 1.05 ± 1.02 | 1.20 ± 1.20 | <0.001 |

Data were n (%) or mean ± SD.
